# Outcome of proliferative lupus nephritis with thrombotic microangiopathy; An ambispective observational single-center study

**DOI:** 10.1186/s12882-025-04154-8

**Published:** 2025-05-13

**Authors:** Ahmed Fayed, Rasmia Elgohary, Amr Mohamed Shaker, Karem Mohamed Salem, Eman El Desouky, Gehad Gamal Maghraby

**Affiliations:** 1 Nephrology Unit, Internal Medicine Department, Kasr Alainy School of Medicine, Cairo University, Cairo, Egypt; 2 Rheumatology and Clinical Immunology Unit, Internal Medicine Department, Kasr Alainy School of Medicine, Cairo University, Cairo, Egypt; 3 Nephrology Unit, Internal Medicine Department, Faculty of Medicine, Fayoum University, Fayoum, Egypt; 4 Department of Epidemiology and Biostatistics, National Cancer Institute, Cairo University, Cairo, Egypt

**Keywords:** Thrombotic microangiopathy, Lupus nephritis, Systemic lupus erythematosus, Plasmapheresis, Cyclophosphamide

## Abstract

**Background:**

Thrombotic microangiopathy (TMA) represents a broad spectrum of diseases. The combination of TMA with lupus nephritis (LN) is associated with worse renal outcomes and a higher mortality rate. To date, there is no agreement on the therapeutic strategies that should be offered to TMA-LN patients.

**Objective:**

In this study, we compared the long-term outcomes of plasma exchange (PLEX) and cyclophosphamide (CYC) in a TMA-LN cohort.

**Methods:**

100 TMA-LN patients who received an induction of steroids and either PLEX or CYC less than 3 months from the start of the study, were selected from the medical records of Kasr Alainy hospitals, Cairo University. The patients were monitored for hematological and renal response at 3, 6, and 12 months.

**Findings:**

In PLEX arm, the mean creatinine level was 1.4 ± 0.7 mg/dl at baseline, decreased to 1.1 ± 0.5 mg/dl after 3 months, and returned to 1.4 ± 1.4 mg/dl at 6 and 12 months (*p* = 0.748). Proteinuria levels significantly decreased from 2.9 ± 0.7 9 gm/24 hrs at baseline to 0.4 ± 0.5 9 gm/24 hrs after 12 months (*p* < 0.001). PLT significantly increased over time with a mean of 65.6 ± 19.0 (x10^₃^)/L at baseline, increasing to 235.9 ± 54.3 (x10^₃^)/L after 12 months (*p* < 0.001). In CYC arm, the mean creatinine level of 1.2 ± 0.6 mg/dl was maintained from baseline through 6 months but significantly increased at 12 months with a mean of 1.9 ± 2.2 mg/dl (*p* = 0.005). Proteinuria levels significantly decreased with means of 3.3 ± 0.6 gm/24 hrs at baseline to 0.7 ± 0.9 gm/24 hrs after 12 months (*p* < 0.001). The PLT increased from 49.5 ± 19.0 (x10^₃^)/L to 198.9 ± 71.5 (x10^₃^)/L after 12 months (*p* < 0.001). At 3- and 12-month intervals, PLEX achieved sustained lower proteinuria (*p* < 0.001 and *p* = 0.047, respectively), higher PLT (*p* < 0.001 and *p* = 0.005, respectively), and higher complement 4 (*p* = 0.001 and *p* < 0.001, respectively), compared to CYC.

**Conclusion:**

Both groups demonstrated significant improvements in renal and hematological outcomes with better long-term renal outcomes in the PLEX arm and comparable improvements in the hematological measures in both groups.

**Supplementary Information:**

The online version contains supplementary material available at 10.1186/s12882-025-04154-8.

## Background

Thrombotic microangiopathy (TMA) is characterized by thrombocytopenia, microangiopathic hemolytic anemia, and microvascular thrombus formation, most commonly affecting the kidneys. It presents either with clinically evident hematological, renal, neurological, and/or gastrointestinal or with isolated renal affection [1]. It represents a disruption in homeostasis involving inflammation, autoimmunity, and complement system abnormalities [2].

While TMA can manifest as primary forms like thrombotic thrombocytopenic purpura (TTP) and hemolytic uremic syndrome (HUS), it also can occur secondary to systemic lupus erythematosus (SLE) [3]. In lupus nephritis (LN), TMA was depicted in 24% of kidney biopsies [4]. TMA in SLE may point to the potential of TTP, complement-mediated HUS, or antiphospholipid syndrome (APS), and may be complicated by additional factors like infection, malignant hypertension, pregnancy, or drugs, which trigger further endothelial damage, and activation of both complement and coagulation pathways [5].

Current guidelines, including the 2018 revised International Society of Nephrology/Renal Pathology Society (ISN/RPS) classification [6], the joint European League Against Rheumatism and European Renal Association-European Dialysis and Transplant Association (EULAR/ERA-EDRA) recommendations [7], and the Kidney Disease Improving Global Outcomes (KDIGO) guidelines [8–10], provide varying approaches to TMA management in LN. Given the high morbidity and mortality associated with TMA in lupus patients [11], early intervention is crucial, with plasma exchange (PLEX) and glucocorticoids recommended as initial management [8, 12]. However, there is limited evidence comparing different therapeutic strategies for TMA in LN.

To date, there is no clear consensus on the management of TMA in patients with LN. The 2018 revised ISN/RPS classification didn’t address the vascular lesions in LN due to insufficient evidence of their prognostic and therapeutic significance [6]. EULAR/ ERA-EDRA guidelines recommended antiphospholipid antibodies (aPL) testing when TMA lesions are present in kidney biopsy raising the suspicion of APS nephropathy [7]. KDIGO guidelines in 2021 and its update in 2024 advocate for targeted treatment based on the clinical context and comprehensive laboratory testing of aPL, a disintegrin-like metalloproteinase with thrombospondin motif type 1 member 13 (ADAMTS 13) activity and anti-ADAMTS 13 antibodies, complement, and genetic studies, though many of these specialized tests face practical limitations in availability and interpretation [8–10]. The high morbidity and mortality associated with TMA in lupus cohort prompts urgent evaluation and intervention [11]. Plasma exchange (PLEX) and glucocorticoids are recommended as the initial early management of TMA in LN while awaiting results [8, 12]. Other options include other immunosuppression therapy targeting LN such as cyclophosphamide (CYC) or rituximab, and those targeting renal microangiopathic APS such as anticoagulation, and considering eculizumab in refractory cases [9, 10].

However, there is limited evidence comparing different therapeutic strategies for TMA management in LN patients. This ambispective study aimed to assess renal outcomes in lupus patients with concomitant proliferative lupus nephritis and thrombotic microangiopathy (TMA-LN), who underwent two different treatment modalities: PLEX and CYC.

## Materials and methods

**Fig. 1:**
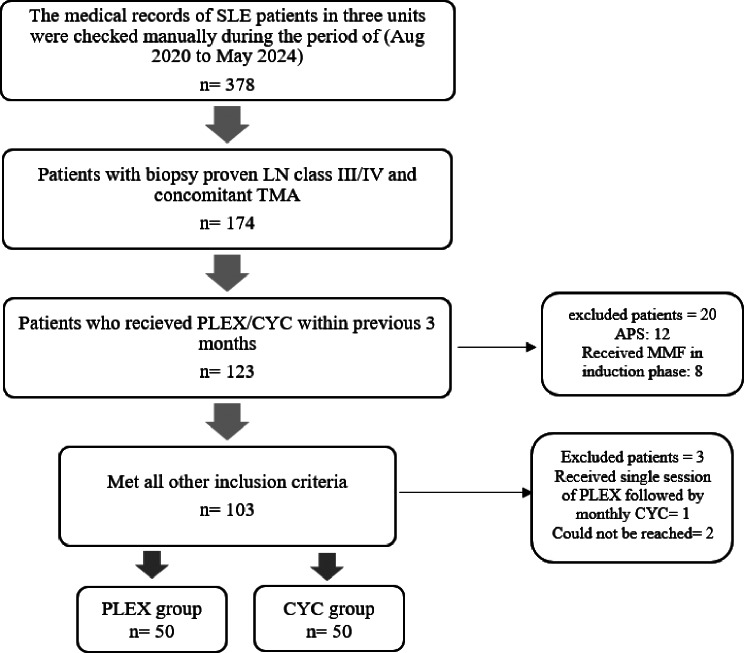
Flowchart of the study. APS: antiphospholipid syndrome, CYC: cyclophosphamide, LN: lupus nephritis, MMF: mycophenolate mofetil, n: number of patients, SLE: systemic lupus erythematosus, TMA: thrombotic microangiopathy

This is an ambispective (retro-prospective) cohort study conducted from August 2020 to May 2024, where each patient was followed for one year after treatment initiation (Fig. 1). The study included 100 SLE patients with LN class III or IV, and concomitant clinical TMA, who underwent either PLEX or CYC in the induction phase of treatment. The diagnosis of SLE fulfilled the 2019 EULAR/ACR classification criteria [13], and LN was diagnosed based on the 2003 ISN/RPS classification system for glomerulonephritis in SLE [14, 15]. Clinical TMA was defined as the presence of microangiopathic hemolytic anemia, fragmented red blood cells, thrombocytopenia, and pathological evidence of thrombotic microangiopathy in renal biopsy, with or without renal impairment. TMA was considered as the presence of glomerular or vascular fibrin thrombi, endothelial swelling or denudation, mesangiolysis, and/or microaneurysms in the glomeruli. The distinction of other features of TMA relative to the lupus class was made by an experienced pathologist [16].

The patients were recruited from the Intensive care, Rheumatology, and Nephrology Units of the Internal Medicine Department at Kasr Alainy Hospital, Cairo University through continuous review of medical records during the study period, we identified and included patients who had received either CYC or PLEX in the induction phase within three months prior to enrollment. Each enrolled patient was then prospectively followed for 12 months from their treatment initiation date. None of the investigators were included in the patients’ treatment plan either during the induction phase or during follow-up visits.

SLE patients without evidence of TMA in their renal biopsy, those with secondary APS as defined by the revised classification criteria for definite antiphospholipid syndrome [17], those who received another therapeutic regimen in the induction phase rather than CYC or PLEX, or those exceeded three months from induction therapy to the time of the study were excluded.

### Baseline assessment

The medical files were reviewed and the following data at admission were extracted: baseline demographic, clinical data, laboratory data including basal metabolic profile, urine analysis, urinary protein in 24 h, aPL, complement 3 (C3), complement 4 (C4), ANA, and anti-dsDNA and pathological data including class of LN, activity index, chronicity index, acute vascular lesions (TMA, vasculitis and vasculopathy) and chronic vascular lesions (intimal fibrosis). Baseline disease activity was assessed by the Systemic Lupus Erythematosus Disease Activity Index 2000 (SLEDAI-2 K) [18]. The type of induction therapeutic approach was documented in every patient. For those who received PLEX, the number of sessions and the exchange volume of plasma were documented.

### Follow-up assessment

The patients were followed up at 3-, 6-, and 12-month intervals from the start of the induction phase. The interval assessment included documentation of any complications with special concern for infection and death, type, and dose of maintenance therapy, as well as laboratory status of kidney functions, serum complements, proteinuria, and the renal response.

### Outcome variables

The primary outcome of this study was an achievement of renal response in each group; defined as preservation or improvement of kidney function accompanied by a reduction in proteinuria at least 25% at 3 months, 50% by 6 months, and proteinuria target below 0.5 gm/24hours by 12 months [7]. Secondary outcomes included improvement of the immunological and hematological parameters (C3, C4, platelets [PLT], lactate dehydrogenase [LDH]) as well as the safety profile of each intervention.

### Ethical statement

The Research Ethics Committee of the Faculty of Medicine, Cairo University, Egypt approved this work (Code: N-215-2023). All procedures performed in this study involving human participants were per the ethical standards of the institutional research committee and with the 1964 Helsinki Declaration. Informed consent was obtained from all individual participants included in the study.

### Statistical methods

Data were analyzed using IBM SPSS advanced statistics (Statistical Package for Social Sciences), version 27 (SPSS Inc., Chicago, IL). Numerical data were described as mean and standard deviation or median and range. Categorical data were described as numbers and percentages. Data was explored for normality using Kolmogorov-Smirnov test and Shapiro-Wilk test. Comparisons between two groups for normally distributed numeric variables were done using the student’s t-test while non-normally distributed numeric variables were done by Mann-Whitney test. Comparisons between categorical variables were performed using the chi-square test, while Fisher’s exact test was used when analyzing subgroups with small, expected frequencies. A p-value of less than or equal to 0.05 was considered statistically significant. All tests were two-tailed. One-way analysis of variance (ANOVA) was used when comparing the same variable at different time points within each studied group. A Post-hoc test was used for multiple comparisons for the same variable at different time points. Multivariate logistic regression analysis was utilized to analyze predictive factors of response.

## Results

The study included 100 SLE patients, their characteristics are presented in Tables 1, 2 and 3. All participants were female with a mean age of 25.19 ± 5.524 years. The mean duration of SLE was 10.21 ± 6.732 months. The mean of baseline SLEDAI score was 13.99 ± 4.781, whereas the activity and chronicity indices from renal biopsy were 13.96 ± 2.526, and 2.50 ± 0.882 respectively. aPL was positive in 23% of the recruited patients.

**Table 1:** Baseline demography, clinical and medication characteristics of both groups

	Group A (PLEX) (*n* = 50)	Group B (CYC) (*n* = 50)	*p* value
**Demographic data** (Mean ± SD)			
Age(years)	25.7 ± 5.8	24 ± 5.6	0.129
Height /cm	156.6 ± 3.5	158.1 ± 3	0.033
Weight /kg	68.2 ± 8.4	66.6 ± 9.1	0.362
BMI (kg/m^2^)	27.8 ± 3.5	26.7 ± 4	0.150
**BMI classification** [Number (%)]			
Normal	9(18.0)	17(34.0)	0.189
Overweight	29(58.0)	23(46.0)	
Obesity	12(24.0)	10(20.0)	
**Clinical data** [Median (IQR)]			
Duration of SLE (months)^*^	7.5(2–30)	10(1–32)	0.108
Duration of LN (months)^*^	4(2–12)	5(1–12)	0.382
Total SLEDAI (mean ± SD)	17.7 ± 2.2	10.2 ± 3.5	< 0.001
**Baseline medications** (Mean ± SD)			
Prednisone (mg/day)	12.4 ± 7.3	10.8 ± 5.7	0.240
Hydroxychloroquine mg/day	200 ± 0	200 ± 0	NA
**Maintenance medications** [Number (%)]			
AZA	18(36.0)	26(52.0)	0.107
Cyclophosphamide	0(0.0)	10 (20.0)	0.001
MMF	32(64.0)	14(28.0)	< 0.001

*p* < 0.05 is statistically significant, AZA: azathioprine, BMI: body mass index, Kg: kilogram, IQR: inter-quartile range, LN: lupus nephritis, mg: milligram, MMF: mycophenolate mofetil, CYC: cyclophosphamide, PLEX: plasma exchange, SD: standard deviation, SLEDAI: Systemic Lupus Erythematosus Disease Activity Index

**Table 2:** Baseline laboratory data of both groups

	Group A (PLEX) (*n* = 50)	Group B (CYC) (*n* = 50)	*p* value
**Laboratory data** (mean ± SD)			
Urea (mg/dL) median (IQR)	37.5(10–182)	30(10–110)	0.638
Creatinine (mg/dL)	1.4 ± 0.7	1.2 ± 0.6	0.208
Uric acid (mg/dL)	5.1 ± 1.1	5.3 ± 1.2	0.402
ALT (U/L) median (IQR)	18(7-170)	20(6–84)	0.958
AST (U/L) median (IQR)	24.5(7-121)	21.5(5–78)	0.170
Hb (gm/L)	10.6 ± 2.9	10.7 ± 3	0.857
WBCs (x10^₃^)/L	7.3 ± 2.1	7.3 ± 2.2	0.944
Platelet (x10^₃^)/L	65.6 ± 19.0	49.5 ± 19.0	< 0.001
LDH (U/L)	873.8 ± 169.3	782.6 ± 157.4	0.006
Fragmented RBCs %	5.8 ± 1.2	4.8 ± 1.2	< 0.001
Total Cholesterol (mg/dL)	186.9 ± 44.2	185.4 ± 34.0	0.850
LDL-c (mg/dL)	118.2 ± 33.7	112.2 ± 28.7	0.339
HDL-c (mg/dL)	48.1 ± 14	44.4 ± 11.1	0.149
TG (mg/dL)	126 ± 58.7	145.2 ± 49.6	0.080
Ca (mg/dL)	8.9 ± 0.6	9 ± 0.6	0.282
Phosphorus (mg/dL)	3.6 ± 0.6	4 ± 0.5	< 0.001
CRP (mg/dL)	5.9 ± 11.2	3.4 ± 4.2	0.137
Urinary protein (gm/24hrs)	2.9 ± 0.7	3.3 ± 0.6	0.015
**Urine sediment**, number (%)			
Free	23(46.0)	0(0.0)	< 0.001
Granular cast	14(28.0)	22(44.0)	
Red cell cast	13(26.0)	28(56.0)	
**Immunological profile**, number (%)			
Serum C3 (mg/dL) (mean ± SD)	82.3 ± 28.4	95.4 ± 41.8	0.071
Serum C4 (mg/dL) (mean ± SD)	11.6 ± 8.7	11.9 ± 8.8	0.904
Anti Cardiolipin IgG (U/ml)	5(10.0)	6(12.0)	0.749
Anti Cardiolipin IgM (U/ml)	6(12.0)	5(10.0)	0.749
Lupus anticoagulant	3(6.0)	3(6.0)	1.000

*p* < 0.05 is statistically significant, ALT: alanine, transaminase, AST: aspartate transaminase, C3: complement 3, C4: complement 4, Ca: calcium, CRP: C-reactive protein, CYC: cyclophosphamide, dL: deciliter, gm: gram, Hb: hemoglobin, HDL-c: high density lipoprotein, IQR: interquartile range, LDH: lactate dehydrogenase, LDL-c: low density lipoprotein, L: liter, mg: milligram, PLEX: plasma exchange, SD: standard deviation, TG: triglycerides, U: unit

**Table 3:** Baseline renal pathology data of both groups

	Group A (PLEX) (*n* = 50)	Group B (CYC) (*n* = 50)	*p* value
Renal Biopsy Class [number (%)]			
a. Class III	24(48.0)	25(50.0)	0.841
b. Class IV	26(52.0)	25(50.0)	
IFTA [number (%)]	2(4.0)	1(2.0)	1.000
Vasculitis [number (%)]	2(4.0)	2(4.0)	1.000
Lupus Vasculopathy [number (%)]	32(64.0)	2(4.0)	1.000
Intimal fibrosis [number (%)]	3(6.0)	3(6.0)	1.000
Activity Index (/24) (mean ± SD)	15.0 ± 2.3	13.0 ± 2.3	< 0.001
Chronicity Index (/12) (mean ± SD)	3.0 ± 0.7	2.0 ± 0.7	< 0.001

*p* < 0.05 is statistically significant, CYC: cyclophosphamide, IFTA: interstitial fibrosis and tubular atrophy, SD: standard deviation, PLEX: plasma exchange

Fifty patients received PLEX in the induction phase as daily sessions with 5% albumin and fresh frozen plasma replacement, 43 patients received one plasma volume, and the other seven patients received one and half plasma volume. After an initial laboratory improvement was observed, the frequency of sessions decreased until treatment was discontinued. The mean number of PLEX sessions was 8.5 ± 1.09. The other fifty patients received CYC according to the National Institute of Health (NIH) protocol at a monthly dose of 15 mg/kg that falls within 0.5-1 gm/m^2^, the originally described doses, for 6 months, not exceeding 1200 mg/dose. The monthly doses were almost stable at a mean of 993 ± 126 mg/dose.

All participants received pulse methylprednisolone 1000 mg daily for the first three days as part of the induction regimen followed by prednisolone 0.6 mg per kg per day orally, which was tapered gradually according to local protocol. Both groups were maintained on immunomodulation therapy after the induction phase. The types of maintenance medication are presented in Table 1. Notably, none of the participants needed dialysis throughout the study.

PLEX group had higher baseline levels of SLEDAI score, LDH, fragmented RBCs, and activity index in renal biopsy (*p* < 0.001, 0.006, < 0.001, and < 0.001 respectively), while CYC group had lower baseline platelet count, and higher proteinuria (*p* < 0.001, 0.015 respectively) (Tables 1, 2 and 3).

Regarding the maintenance medications, the PLEX group frequently used mycophenolate mofetil (MMF), followed by azathioprine (AZA). In contrast, in the CYC group, AZA was more common, with MMF being the second most frequent choice. Moreover, 10 patients in CYC group received six additional CYC doses at three months intervals as maintenance therapy (Table 1).

### Renal outcomes

**Fig. 2:**
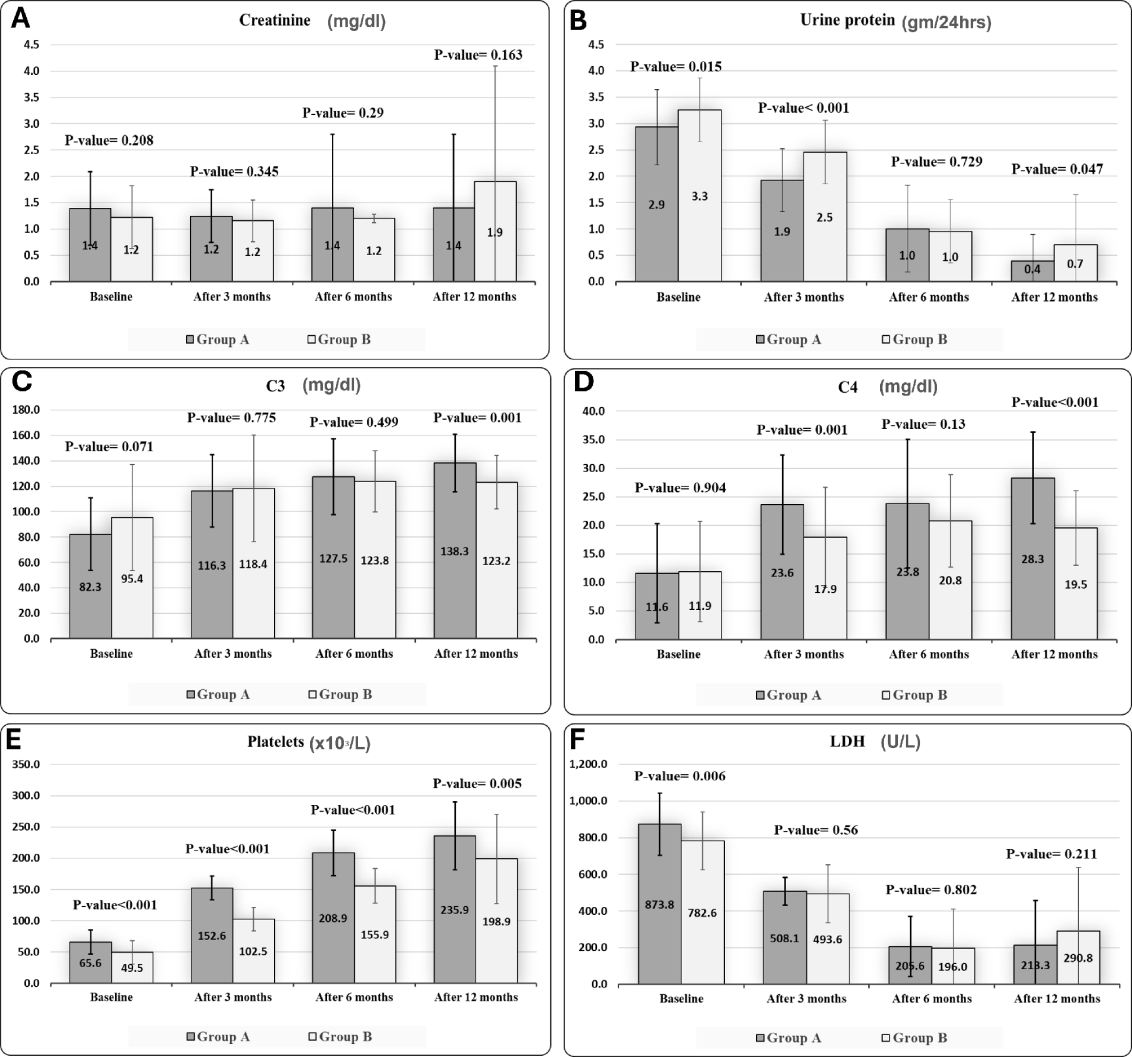
Comparison between PLEX and CYC groups regarding urinary proteins in 24 h (**A**) serum creatinine (**B**) serum complement C3 (**C**) complement C4 (**D**) platelet count (**E**) and serum LDH (**F**) at baseline, in addition at 3 months, 6 months, and 12 months after the induction phase. PLEX: plasma exchange, CYC: cyclophosphamide, LDH: lactate dehydrogenase

The changes in mean creatinine levels were not significant between both groups (Fig. 2-A, Table S-1). However, they showed different patterns of temporal changes in each group. In the PLEX group, mean creatinine levels were 1.4 ± 0.7 mg/dl at baseline, decreased to 1.1 ± 0.5 mg/dl at 3 months, and returned to 1.4 ± 1.4 mg/dl at 6 and 12 months (*p* = 0.748) (Table S-2). Pairwise comparisons revealed no significant differences across time points (Table S-3). In contrast, the CYC group maintained stable levels of creatinine at 1.2 ± 0.6 mg/dl from baseline to 6 months but showed a significant increase to 1.9 ± 2.2 mg/dl at 12 months (*p* = 0.005) (Table S-2). Pairwise comparisons revealed significant increases at 12 months compared to baseline (*p* = 0.005), 3 months (*p* = 0.002), and 6 months (*p* = 0.003) (Table S-3).

Proteinuria improved significantly in both groups at 12 months compared to baseline. In the PLEX group, the proteinuria decreased from 2.9 ± 0.7 gm/24 hrs to 0.4 ± 0.5 gm/24 hrs, while in the CYC group, it decreased from 3.3 ± 0.6 gm/24 hrs to 0.7 ± 0.9 gm/24 hrs (both *p* < 0.001; Table S-4). Notably, the PLEX group achieved a significantly greater reduction in proteinuria at 3 months (*p* < 0.001) and maintained lower levels at 12 months (*p* = 0.047) compared to CYC (Fig. 2-B, Table S-1). Pairwise comparisons showed significant differences between all-time points in the PLEX group. In contrast, the CYC group showed significant reductions in proteinuria between baseline and 3 months, and between 3 and 6 months (*p* < 0.001), but the change from 6 to 12 months was not significant (*p* = 0.077; Table S-5).

**Fig. 3:**
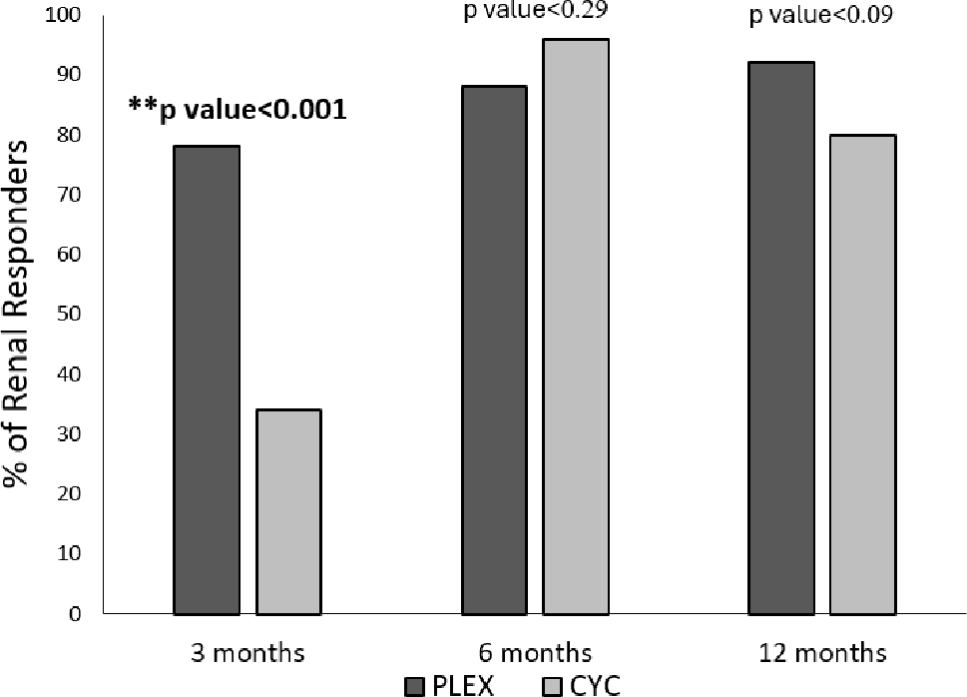
Comparison between PLEX and CYC groups regarding the % of renal responders at 3 months, 6 months, and 12 months after the induction phase. PLEX: plasma exchange, CYC: cyclophosphamide, **: statistically significant

At 3 months, more patients in the PLEX group significantly achieved early renal response compared to the CYC group (78% vs. 30%, *p* = 0.001). While both groups improved over time, the between-group differences became non-significant at later time points. The proportion of patients achieving partial renal response at 6 months (PLEX 88% vs. CYC 96%, *p* = 0.296) and complete renal response at 12 months (PLEX 91.7% vs. CYC 79%, *p* = 0.091) was similar between groups (Fig. 3).

### Immunological outcomes

Both groups showed significant improvement in C3 levels from baseline compared to each of the three time points (*p* < 0.001; Tables S-6, S-7). In the PLEX group, C3 improvements plateaued between 6 and 12 months, whereas the CYC group showed consistent, nonsignificant changes across all time points (Table S-7). Comparisons between both groups showed no significant differences at the 3 and 6 months (*p* = 0.77, 0.49 respectively; Fig. 2-C), but the PLEX group demonstrated significantly higher C3 levels at 12 months compared to the CYC group (*p* = 0.001; Fig. 2-C).

Both groups also showed improvement in C4 levels from baseline compared to all time points (Tables S-8, S-9). The PLEX group demonstrated significant C4 improvement between 3 and 12 months (*p* = 0.015) and between 6 and 12 months (*p* = 0.019), but not between 3 and 6 months (*p* = 0.919) (Table S-9). In contrast, the CYC group showed no significant differences between any time points (Table S-9). Between-group comparisons revealed significantly higher C4 levels in the PLEX group at both 3 months (*p* = 0.001) and 12 months (*p* < 0.001) compared to CYC (Fig. 2-D, Table S-1).

### Hematological outcomes

Both treatments led to significant improvements in platelet count and LDH levels from baseline through 12 months (*p* < 0.001) (Table S-10, S-11). Platelet count in the PLEX group increased from 65.6 ± 19.0 (x10^₃^)/L to 235.9 ± 54.3 (x10^₃^)/L, while in the CYC group they rose from 49.5 ± 19.0 (x10^₃^)/L to 198.9 ± 71.5 (x10^₃^)/L at 12 months (Table S-10). Between-group comparisons showed significantly higher platelets recovery in the PLEX group at all time points: 3 months (*p* < 0.001), 6 months (*p* < 0.001), and 12 months (*p* = 0.005) (Fig. 2-E, Table S-1).

LDH levels also decreased in both groups from baseline to 12 months: PLEX group from 873.8 ± 169.3 U/L to 213.3 ± 244.9 U/L, and CYC group from 782.6 ± 157.4 U/L to 290.8 ± 348.1 U/L. The PLEX group showed a significant LDH reduction between 3 and 6 months, but no significant change between 6 and 12 months (Table S-11). The CYC group demonstrated significant improvements across all time points (Table S-11) and achieved significantly lower LDH levels compared to PLEX at both 6 months (*p* = 0.026) and 12 months (*p* = 0.007) (Fig. 2-F, Table S-1).

### Predictive factors of renal response in each group

In multivariate logistic regression analysis, none of the following factors significantly predicted renal response at 6 or 12 months in either treatment group: age, aPL positivity, duration of SLE, duration of LN, class of LN, activity index, chronicity index, and SLEDAI score (Tables S-14 to S-17).

### Impact of number and volume of PLEX sessions on patients’ response

The improvement noticed in the PLEX group was not associated with the number of sessions (Table S-18). However, some parameters were associated with exchanged volume at the time of induction (Table S-19).

### Interventions- related complications

Regarding early complications after intervention (Table S-1), in the PLEX group, hypotension, hypocalcemia, and catheter-related infections occurred in eight, six, and three patients respectively. In the CYC group, mild leucopenia occurred in four patients requiring no specific intervention, and two patients developed pneumonia. At 3, 6, and 12-month follow-ups (Table S1), there were no statistically significant differences between PLEX and CYC groups in infection, disease flare, leucopenia, or death.

## Discussion

In this ambispective observational study, we evaluated outcomes of TMA-LN in patients treated with either PLEX or CYC in addition to pulse steroids during the induction phase, followed by maintenance immunosuppression. Both treatment protocols demonstrated significant improvements in renal, immunological, and hematological outcomes over 12 months, though with notable differences in response patterns.

Both treatment groups presented with high mean SLEDAI scores and renal activity indices, which have been previously established as major risk factors for TMA in LN patients [19], Despite this disease severity, none of the patients in our study required dialysis. The differences in maintenance therapy between groups, predominantly MMF in the PLEX group versus AZA in the CYC group, were largely attributable to a period of MMF unavailability in our country during part of the study. During this time, more patients received CYC induction, and AZA was the primary maintenance option. This also explains why a small number of patients in the CYC group continued CYC as a maintenance therapy.

Regarding short-term follow-up, the PLEX group showed superior renal and hematological outcomes at 3 months, with significantly lower proteinuria, greater achievement of partial renal response, higher C3 and C4 levels, higher platelet count, and more reduction in LDH levels.

This early advantage of PLEX aligns with its mechanism of action, which provides immediate removal of pathogenic autoantibodies (including those against ADAMTS13 and complement regulators), mutant complement proteins, procoagulant factors, and immune complexes. Furthermore, PLEX replaces deficient or mutated circulating ADAMTS13 and complement regulators, thereby addressing multiple potential mechanisms underlying TMA in SLE patients [20].

Momtaz et al. (2018) reported total renal recovery in 50% of patients and partial improvement in 29% among fourteen SLE patients with limited renal TMA who received PLEX in addition to standard immunosuppressive therapy. Consistent with our results, they observed early renal recovery after 6 to 8 PLEX sessions [21]. Additionally, studies reported that patients with TMA-LN who received PLEX and immunomodulatory drugs were able to discontinue renal replacement therapy [22, 23].

Chen et al. similarly reported significantly higher C3 levels immediately and 2 months after PLEX therapy in 42 TMA-LN patients. However, contrary to our results, they demonstrated no significant increase in platelet count [24].

In contrast, the French National TMA registry, which included 18 SLE patients among other connective tissue diseases, reported that secondary TMA responded well to immunosuppressive treatment alone, with complete remission achieved in most cases. PLEX did not demonstrate an early effect on TMA features on either Day 7 or Day 15. It is worth noting that, despite the heterogeneity of the included population and their varied presentations, the analysis was conducted across all cases without sufficient distinction. This, along with the limited sample size of lupus patients, may account for the discrepancy with our findings [25].

Another possible explanation for differences in treatment response comes from differences in the pathophysiological mechanisms in TMA. Notably, Park et al. presented 11 cases of TMA-LN refractory to PLEX, glucocorticoids, and immune-modulatory therapies. Ten of 11 patients (91%) responded favorably to eculizumab, complement-targeted therapy, with significant improvements in both renal outcomes and hematological parameters. These patients demonstrated laboratory evidence of alternative complement pathway activation with decreased C3 levels but normal C4 levels, distinguishing them from classic lupus-mediated hypocomplementemia. Renal biopsies showed prominent C5b-9 (membrane attack complex) deposition in the microvasculature, supporting complement-mediated endothelial injury. While PLEX non-specifically removes plasma components (including both pathogenic and protective factors), eculizumab specifically targets terminal complement activation at C5, directly addressing the pathogenic mechanism evidenced by the C5b-9 deposition [26].

On long-term follow-up, both groups experienced similar rates of partial renal response and reduction in proteinuria at 6 months, while the CYC group exhibited lower creatinine levels at this time point. At 12 months, 91.7% of patients in the PLEX group achieved a complete renal response compared to 79.6% in the CYC group. However, this difference did not reach statistical significance. Patients in the PLEX group maintained stable creatinine levels, while those in the CYC group showed increased creatinine levels, which suggests potentially better long-term preservation of the renal function with PLEX. Regarding the hematological outcomes, both treatment protocols achieved remarkable platelets recovery and decreased LDH levels, indicating effective control of microangiopathic hemolysis. However, the PLEX group demonstrated consistently higher platelet count at all follow-up time points.

These findings should be interpreted carefully due to the recognized short-term effect of PLEX, and the fact that 64% of PLEX arm was maintained on MMF compared to only 28% in the CYC arm. We assumed that using PLEX in the induction phase may achieve rapid control of disease activity, providing a platform for immunomodulation therapy to sustained remission. While analyzing the specific impact of different maintenance therapy combinations would provide valuable insights, such analysis proved challenging in our study due to variations in dosing regimens and escalation protocols across patients. This represents an important point for future research.

Our findings are consistent with the retrospective study by Li et al. who demonstrated that TMA-LN patients who received PLEX in addition to conventional immunosuppressive therapy had significantly better renal outcomes compared to those treated with immunosuppression alone. Plasmapheresis treatment was associated with higher rates of complete renal remission, lower progression to end-stage renal disease, and better preservation of renal function over a 3-year period [23]. Additionally, PLEX was found to be associated with a significant reduction of lupus activity, creatinine level, and improvement of vascular endothelial dysfunction markers [27]. Notably, the retrospective study conducted by Hu et al. found that 60% of patients with TMA-LN who received prednisone and MMF during induction and did not require renal replacement therapy, exhibited a favorable renal response [22].

However, a prospective study by Pattanashetti et al. found no significant benefits from adding PLEX to standard therapy in TMA-LN patients. Their findings contrast with our study, likely due to important methodological differences, only eight of their 50 TMA-LN patients received PLEX, and these patients underwent just five sessions, which may have been insufficient to achieve optimal therapeutic effect [28].

There remains a lack of standardized protocols regarding the optimal number of PLEX sessions, frequency, and total volume. Current practices are largely extrapolated from thrombotic thrombocytopenic purpura (TTP) management protocols [29, 30]. Our study utilized a protocol of 6–11 sessions, which may have contributed to the favorable outcomes observed, as fewer sessions have shown less consistent results [28].

Regarding cyclophosphamide, contrary to our results, Hu et al. suggested that CYC alone is often insufficient to control the TMA process in lupus nephritis [22].

In our study, we found no predictive factors for renal response achievement in either group, suggesting that both treatment protocols can be effective regardless of baseline patient characteristics. This differs from Momtaz et al., who reported that renal improvement was significantly negatively correlated with pre-PLEX serum creatinine levels [21].

Regarding safety, our study showed comparable rates of disease flares and complications, including infections, leukopenia, and mortality, between the treatment groups, indicating similar safety profiles. This observation aligns with several studies that have reported acceptable safety profiles for both PLEX and cyclophosphamide in lupus patients [31, 32].

Compared to the poor prognoses previously reported for TMA-LN patients, our study demonstrated better renal outcomes across both treatment groups. This improvement may be attributed to early diagnosis and prompt intervention. In addition, none of the included patients required acute dialysis, which is a recognized risk factor for progression to end-stage renal disease [22]. Moreover, our patients had favorable baseline histopathological characteristics in renal biopsies, with high activity indices, low chronicity indices, and limited presence of intimal fibrosis (only 6% of biopsies), which has been associated with better treatment response [33].

### Strengths and limitations

The study has several strengths: it included a reasonably large sample size of 100 SLE patients to draw meaningful conclusions, comprehensive baseline data collection that provided a thorough characterization of the patient population, systematic short and long-term follow-up at 3, 6, and 12 months, and finally, the investigators were independent and not involved in the patients’ treatment plans, minimizing potential bias.

However, there are some limitations that should be considered. Being an observational ambispective study, our findings cannot establish causality with the certainty of a randomized controlled trial (RCT). However, our study provides valuable real-world evidence of treatment patterns and outcomes in this specific patient population. Also, the study was conducted at a single center, however, patients were recruited from three major specialized units (Intensive Care, Rheumatology, and Nephrology) at Kasr Alainy Hospital, Cairo University, a national referral center serving patients from across the country. This diverse patient population and varied clinical settings enhance the generalizability of our findings, though results should still be interpreted within the context of a single-center study.

The induction and maintenance treatment strategies in this study were determined by each treating unit’s established protocols, treating physician judgment, and local medication availability. While this reflects a real clinical practice, we acknowledge that this treatment heterogeneity poses challenges for results interpretation and reproducibility. The variation in treatment approaches reflects the current lack of standardization in TMA-LN management, highlighting the critical need for more evidence to guide treatment decisions. Despite the comprehensive baseline data collection, there were unmeasured confounding factors that could influence the selection of the induction protocol and hence may impact the outcomes, such as ADAMTS 13 activity and antibody levels, complement factor H antibodies, or complement genetic mutation tests, which were not available at the study center at the time of the study.

## Conclusion

The current study demonstrated that both PLEX and CYC treatment protocols have merit. PLEX intervention might have better long-term renal outcomes and sustained immunological response when followed by maintenance of immunosuppression. Both treatment modalities had comparable improvements in the hematological measures. Both groups had similar rates of flare and complications, including infection, leucopenia, and death. Accordingly, PLEX could be considered as a potential induction therapy option for SLE patients with TMA-LN. Further multicenter RCTs are recommended before generalizing these results.

## Electronic supplementary material

Below is the link to the electronic supplementary material.

Supplementary Material 1

## Data Availability

The data that support the findings of this study are available from the corresponding author upon reasonable request.

## Abbreviations

ADAMTS 13: A disintegrin-like metalloproteinase with thrombospondin motif type 1 member 13
aPL: Anti-phospholipid antibodies
APS: Antiphospholipid syndrome
AZA: Azathioprine
C3: Complement 3
C4: Complement 4
CYC: Cyclophosphamide
EULAR/ ERA-EDRA: The joint European League Against Rheumatism and European Renal Association-European Dialysis and Transplant Association
HUS: Hemolytic uremic syndrome
ISN/RPS: International Society of Nephrology/Renal Pathology Society
KDIGO: Kidney Disease Improving Global Outcomes
LDH: Lactate dehydrogenase
MMF: Mycophenolate mofetil
NIH: National Institute of Health
PLEX: Plasma exchange
PLT: Platelet count
RCTs: Randomized controlled clinical trials
SLE: Systemic lupus erythematosus
SLEDAI: Systemic Lupus Erythematosus Disease Activity Index
TMA: Thrombotic microangiopathy
TMA-LN: Concomitant proliferative lupus nephritis and thrombotic microangiopathy
TTP: Thrombotic thrombocytopenic purpura
