# Supplementary material for: Outcome of proliferative lupus nephritis with thrombotic microangiopathy; An ambispective observational single-center study
Source: BMC Nephrol. 2025 May 13;26:233. doi: 10.1186/s12882-025-04154-8 (PMC12076891; doi:10.1186/s12882-025-04154-8)
Supplement: Supplementary file 1 — Supplementary Material 1 [file 12882_2025_4154_MOESM1_ESM.docx]

| **Table S-1: Comparison Between the Two Groups Regarding Laboratory Response and Complications Across Different Time Points** | | | | |
| --- | --- | --- | --- | --- |
|  |  | **Group A (PLEX)** | **Group B (CYC)** | **p value** |
| **Immediate complications (within 2 weeks from induction), number (%)** | |  |  |  |
|  | Hypotension | 8(16) |  |  |
|  | Hypocalcemia | 6(12) |  |  |
|  | Infection | 3(6) **[Infected Catheter]** | 2(4) **[pneumonia]** |  |
|  | Leukopenia | 0 | 4 (8) |  |
| **3 months** | Creatinine, Mean ± SD | 1.1±0.5 | 1.2±0.4 | 0.345 |
|  | Urinary protein (gm/24hrs), Mean ± SD | 1.9±0.6 | 2.5±0.6 | <0.001 |
|  | Complement- C3, Mean ± SD | 116.3±28.4 | 118.4±41.8 | 0.775 |
|  | Complement C4, Mean ± SD | 23.6±8.7 | 17.9±8.8 | 0.001 |
|  | Platelet count, Mean ± SD | 152.6±19.0 | 102.5±19.0 | <0.001 |
|  | LDH, Mean ± SD | 508±75 | 494±158 | 0.56 |
|  | Renal response- 25% reduction, number (%) | 39(78.0) | 17(34.0) | <0.001 |
|  | Complications, number (%) | 0 | 0 |  |
| **6 months** | Creatinine, Mean ± SD | 1.4±1.4 | 1.2±0.8 | 0.29 |
|  | Urinary protein (gm/24hrs), Mean ± SD | 1.0±0.8 | 1±0.6 | 0.729 |
|  | Complement- C3, Mean ± SD | 127.5±29.8 | 123.8±24.2 | 0.499 |
|  | Complement C4, Mean ± SD | 23.8±11.3 | 20.8±8.1 | 0.130 |
|  | Platelet count, Mean ± SD | 208.9±36.2 | 155.9±27.6 | <0.001 |
|  | LDH, Mean ± SD | 205.6±163.1 | 196±216.5 | 0.802 |
|  | Renal response- 50% reduction, number (%) | 44(88.0) | 48(96.0) | 0.296 |
|  | Complications, number (%) |  |  |  |
|  | Infection | 3(6) | 4(8) | 1.000 |
|  | Leukopenia | 6(12) | 6(12) | 1.000 |
|  | Flare | 3(6) | 2(4) | 1.000 |
|  | Mortality | 2(4) | 2(4) | 1.000 |
| **12 months** | Creatinine, Mean ± SD | 1.4±1.4 | 1.9±2.2 | 0.163 |
|  | Urinary protein (gm/24hrs), Mean ± SD | 0.4±0.5 | 0.7±0.9 | 0.047 |
|  | Complement- C3, Mean ± SD | 138.3±22.6 | 123.2±21.1 | 0.001 |
|  | Complement C4, Mean ± SD | 28.3±8.0 | 19.5±6.5 | <0.001 |
|  | Platelet count, Mean ± SD | 235.9±54.3 | 198.9±71.5 | 0.005 |
|  | LDH, Mean ± SD | 213.3±244.9 | 290.8±348.1 | 0.211 |
|  | Complete renal response, number (%) | 44(91.7) | 39(79.6) | 0.091 |
|  | Complications, number (%) |  |  |  |
|  | Infection | 4(8.3) | 9(18.8) | 0.136 |
|  | Leukopenia | 4(8.3) | 9(18.8) | 0.136 |
|  | Flare | 4(8.3) | 9(18.8) | 0.136 |
|  | Mortality | 4(8.3) | 9(18.8) | 0.136 |
| p<0.05 is statistically significant, CYC: cyclophosphamide, gm: gram, IQR: inter-quartile range, LDH: lactate dehydrogenase, PLEX: plasma exchange, SD: standard deviation. | | | | |

| **Table S-2: Temporal Changes in Serum Creatinine in Each Group** | | | | | |
| --- | --- | --- | --- | --- | --- |
| **Creatinine** | **Baseline** | **After 3 months** | **After 6 months** | **After 12 months** | **p value** |
|  | **Mean ±SD** | **Mean ± SD** | **Mean ±SD** | **Mean ±SD** |  |
| **PLEX** | 1.4±0.7 | 1.1±0.5 | 1.4±1.4 | 1.4±1.4 | 0.748 |
| **CYC** | 1.2±0.6 | 1.2±0.4 | 1.2±0.8 | 1.9±2.2 | **0.005** |
| p<0.05 is statistically significant, CYC: cyclophosphamide, PLEX: plasma exchange, SD: standard deviation. | | | | | |

| **Table S-3: Pairwise Comparison of Serum Creatinine Across Time points in Each Group** | | | | |
| --- | --- | --- | --- | --- |
| **Serum Creatinine** | **PLEX** | | **CYC** | |
|  | **LSD** | **p value** | **LSD** | **p value** |
| Baseline Vs After 3 months | 0.144 | 0.499 | 0.070 | 0.772 |
| Baseline Vs After 6 months | -0.030 | 0.888 | 0.048 | 0.843 |
| Baseline Vs After 12 months | 0.001 | 0.998 | -0.686 | **0.005** |
| After 3 months Vs After 6 months | -0.174 | 0.414 | -0.022 | 0.927 |
| After 3 months Vs After 12 months | -0.144 | 0.505 | -0.756 | **0.002** |
| After 6 months Vs After 12 months | 0.031 | 0.887 | -0.734 | **0.003** |
| p<0.05 is statistically significant, CYC: cyclophosphamide, LSD: least significant difference, PLEX: plasma exchange, Vs: versus. | | | | |

| **Table S-4: Temporal Changes of Proteinuria in Each Group.** | | | | | |
| --- | --- | --- | --- | --- | --- |
| **Urine Protein** | **Baseline** | **After 3 months** | **After 6 months** | **After 12 months** | **p value** |
|  | **Mean ±SD** | **Mean ± SD** | **Mean ±SD** | **Mean ±SD** |  |
| **PLEX** | 2.9±0.7 | 1.9±0.6 | 1.0±0.8 | 0.4±0.5 | **<0.001** |
| **CYC** | 3.3±0.6 | 2.5±0.6 | 1±0.6 | 0.7±0.9 | **<0.001** |
| p<0.05 is statistically significant, CYC: cyclophosphamide, PLEX: plasma exchange, SD: standard deviation. | | | | | |

| **Table S-5: Pairwise Comparison of Proteinuria Across Time points in Each Group** | | | | |
| --- | --- | --- | --- | --- |
| **Proteinuria** | **PLEX** | | **CYC** | |
|  | **LSD** | **p value** | **LSD** | **p value** |
| Baseline Vs After 3 months | 1.006 | **<0.001** | 0.800 | **<0.001** |
| Baseline Vs After 6 months | 1.926 | **<0.001** | 2.304 | **<0.001** |
| Baseline Vs After 12 months | 2.540 | **<0.001** | 2.556 | **<0.001** |
| After 3 months Vs After 6 months | 0.920 | **<0.001** | 1.504 | **<0.001** |
| After 3 months Vs After 12 months | 1.534 | **<0.001** | 1.756 | **<0.001** |
| After 6 months Vs After 12 months | 0.614 | **<0.001** | 0.252 | 0.077 |
| p<0.05 is statistically significant, CYC: cyclophosphamide, LSD: least significant difference, PLEX: plasma exchange, Vs: versus. | | | | |

| **Table S-6: Temporal Changes of Serum Complement C3 in Each Group.** | | | | | |
| --- | --- | --- | --- | --- | --- |
| **C3** | **Baseline** | **After 3 months** | **After 6 months** | **After 12 months** |  |
|  | **Mean ±SD** | **Mean ± SD** | **Mean ±SD** | **Mean ±SD** | **p value** |
| **PLEX** | 82.3±28.4 | 116.3±28.4 | 127.5±29.8 | 138.3±22.6 | **<0.001** |
| **CYC** | 95.4±41.8 | 118.4±41.8 | 123.8±24.2 | 123.2±21.1 | **<0.001** |
| p<0.05 is statistically significant, C3: complement 3, CYC: cyclophosphamide, PLEX: plasma exchange, SD: standard deviation. | | | | | |

| **Table S-7: Pairwise Comparison of Serum Complement C3 Across Time points in Each Group** | | | | |
| --- | --- | --- | --- | --- |
| **C3** | **PLEX** | | **CYC** | |
|  | **LSD** | **p value** | **LSD** | **p value** |
| Baseline Vs After 3 months | -34.000 | **<0.001** | -23.000 | **0.001** |
| Baseline Vs After 6 months | -45.212 | **<0.001** | -28.484 | **<0.001** |
| Baseline Vs After 12 months | -56.003 | **<0.001** | -27.830 | **<0.001** |
| After 3 months Vs After 6 months | -11.212 | **0.043** | -5.484 | 0.418 |
| After 3 months Vs After 12 months | -22.003 | **<0.001** | -4.830 | 0.480 |
| After 6 months Vs After 12 months | -10.791 | 0.054 | 0.655 | 0.924 |
| p<0.05 is statistically significant, Complement 3: C3, CYC: cyclophosphamide, LSD: least significant difference, PLEX: plasma exchange, Vs: versus. | | | | |

| **Table S-8: Temporal Changes of Serum Complement C4 in Each Group** | | | | | |
| --- | --- | --- | --- | --- | --- |
| **C4** | **Baseline** | **After 3 months** | **After 6 months** | **After 12 months** |  |
|  | **Mean ±SD** | **Mean ± SD** | **Mean ±SD** | **Mean ±SD** | **p value** |
| **PLEX** | 11.6±8.7 | 23.6±8.7 | 23.8±11.3 | 28.3±8.0 | **<0.001** |
| **CYC** | 11.9±8.8 | 17.9±8.8 | 20.8±8.1 | 19.5±6.5 | **<0.001** |
| p<0.05 is statistically significant, C4: complement 4, CYC: cyclophosphamide, PLEX: plasma exchange, SD: standard deviation. | | | | | |

| **Table S-9: Pairwise Comparison of Serum Complement C4 Across Time points in Each Group** | | | | |
| --- | --- | --- | --- | --- |
| **C4** | **PLEX** | | **CYC** | |
|  | **LSD** | **p value** | **LSD** | **p value** |
| Baseline Vs After 3 months | -12.000 | **<0.001** | -6.000 | **<0.001** |
| Baseline Vs After 6 months | -12.190 | **<0.001** | -8.982 | **<0.001** |
| Baseline Vs After 12 months | -16.610 | **<0.001** | -7.606 | **<0.001** |
| After 3 months Vs After 6 months | -0.190 | 0.919 | -2.982 | 0.068 |
| After 3 months Vs After 12 months | -4.610 | **0.015** | -1.606 | 0.328 |
| After 6 months Vs After 12 months | -4.420 | **0.019** | 1.376 | 0.402 |
| p<0.05 is statistically significant, Complement 4: C4, CYC: cyclophosphamide, LSD: least significant difference, PLEX: plasma exchange, Vs: versus. | | | | |

| **Table S-10: Temporal Changes of Platelets Count in Each Group.** | | | | | |
| --- | --- | --- | --- | --- | --- |
| **PLTs** | **Baseline** | **After 3 months** | **After 6 months** | **After 12 months** |  |
|  | **Mean ±SD** | **Mean ± SD** | **Mean ±SD** | **Mean ±SD** | **p value** |
| **PLEX** | 65.6±19.0 | 152.6±19.0 | 208.9±36.2 | 235.9±54.3 | **<0.001** |
| **CYC** | 49.5±19.0 | 102.5±19.0 | 155.9±27.6 | 198.9±71.5 | **<0.001** |
| p<0.05 is statistically significant, CYC: cyclophosphamide, PLEX: plasma exchange, PLTs: platelet count, SD: standard deviation. | | | | | |

| **Table S-11: Pairwise Comparison of Platelet Counts Across Time points in Each Groups** | | | | |
| --- | --- | --- | --- | --- |
| **Platelets Count** | **PLEX** | | **CYC** | |
|  | **LSD** | **p value** | **LSD** | **p value** |
| Baseline Vs After 3 months | -87.000 | **<0.001** | -53.000 | **<0.001** |
| Baseline Vs After 6 months | -143.320 | **<0.001** | -106.320 | **<0.001** |
| Baseline Vs After 12 months | -170.338 | **<0.001** | -149.398 | **<0.001** |
| After 3 months Vs After 6 months | -56.320 | **<0.001** | -53.320 | **<0.001** |
| After 3 months Vs After 12 months | -83.338 | **<0.001** | -96.398 | **<0.001** |
| After 6 months Vs After 12 months | -27.018 | **<0.001** | -43.078 | **<0.001** |
| p<0.05 is statistically significant, CYC: cyclophosphamide, LSD: least significant difference, PLEX: plasma exchange, Vs: versus. | | | | |

| **Table S-12: Temporal Changes of Serum LDH in Each Group.** | | | | | |
| --- | --- | --- | --- | --- | --- |
| **LDH** | **Baseline** | **After 3 months** | **After 6 months** | **After 12 months** |  |
|  | **Mean ±SD** | **Mean ± SD** | **Mean ±SD** | **Mean ±SD** | **p value** |
| **PLEX** | 873.8±169.3 | 508.1±75.5 | 205.6±163.1 | 213.3±244.9 | **<0.001** |
| **CYC** | 782.6±157.4 | 493.6±157.4 | 196±216.5 | 290.8±348.1 | **<0.001** |
| p<0.05 is statistically significant, CYC: cyclophosphamide, LDH: lactate dehydrogenase, PLEX: plasma exchange, SD: standard deviation. | | | | | |

| **Table S-13: Pairwise Comparison of Serum LDH Across Time points in Each Group** | | | | |
| --- | --- | --- | --- | --- |
| **LDH** | **PLEX** | | **CYC** | |
|  | **LSD** | **p value** | **LSD** | **p value** |
| Baseline Vs After 3 months | 365.740 | **<0.001** | 289.000 | **<0.001** |
| Baseline Vs After 6 months | 668.200 | **<0.001** | 586.660 | **<0.001** |
| Baseline Vs After 12 months | 660.467 | **<0.001** | 491.869 | **<0.001** |
| After 3 months Vs After 6 months | 302.460 | **<0.001** | 297.660 | **<0.001** |
| After 3 months Vs After 12 months | 294.727 | **<0.001** | 202.869 | **<0.001** |
| After 6 months Vs After 12 months | -7.733 | 0.825 | -94.791 | **0.044** |
| p<0.05 is statistically significant, CYC: cyclophosphamide, LDH: lactate dehydrogenase, LSD: least significant difference, PLEX: plasma exchange, Vs: versus. | | | | |

| **Table S-14: Multivariate Logistic Regression Analysis for Studied Data as A Predictive Factor of Partial Renal Response At 6 Months In PLEX Group.** | | | | | | |
| --- | --- | --- | --- | --- | --- | --- |
|  | **B** | **SE** | **p-value** | **Odds** | **95% CI** | |
| **Age** | 0.026 | 0.080 | 0.749 | 1.026 | 0.877 | 1.201 |
| **APL positivity** | -0.665 | 0.943 | 0.481 | 0.514 | 0.081 | 3.267 |
| **Duration of SLE** | 0.065 | 0.083 | 0.432 | 1.067 | 0.907 | 1.256 |
| **Duration of LN** | 0.158 | 0.211 | 0.452 | 1.172 | 0.775 | 1.771 |
| **Class of LN** | -1.700 | 1.136 | 0.135 | 0.183 | 0.020 | 1.693 |
| **Activity index** | 0.129 | 0.183 | 0.481 | 1.138 | 0.795 | 1.629 |
| **Chronicity index** | 0.369 | 0.614 | 0.549 | 1.446 | 0.434 | 4.820 |
| **SLEDAI score** | 0.200 | 0.233 | 0.389 | 1.222 | 0.775 | 1.927 |
| APL: antiphospholipid antibodies, B: Regression coefficient, CI: Confidence interval, LN: lupus nephritis, PLEX: plasma exchange, SE: Standard error, SLE: systemic lupus erythematosus, SLEDAI: Systemic Lupus Erythematosus Disease Activity Index. | | | | | | |

| **Table S-15: Multivariate logistic regression analysis for studied data as a predictive factor of complete renal response at 12 months in PLEX group** | | | | | | |
| --- | --- | --- | --- | --- | --- | --- |
|  | **B** | **SE** | **p-value** | **Odds** | **95% CI** | |
| **Age** | -0.013 | 0.088 | 0.880 | 0.987 | 0.830 | 1.174 |
| **APL positivity** | -0.125 | 1.209 | 0.918 | 0.882 | 0.082 | 9.443 |
| **Duration of SLE** | 0.040 | 0.091 | 0.656 | 1.041 | 0.872 | 1.244 |
| **Duration of LN** | -0.031 | 0.190 | 0.869 | 0.969 | 0.668 | 1.406 |
| **Class of LN** | -1.190 | 1.193 | 0.319 | 0.304 | 0.029 | 3.157 |
| **Activity index** | 0.180 | 0.214 | 0.400 | 1.198 | 0.787 | 1.824 |
| **Chronicity index** | 1.122 | 0.850 | 0.187 | 3.072 | 0.580 | 16.260 |
| **SLEDAI score** | 0.277 | 0.298 | 0.352 | 1.319 | 0.736 | 2.366 |
| APL: antiphospholipid antibodies, B: Regression coefficient, CI: Confidence interval, LN: lupus nephritis, PLEX: plasma exchange, SE: Standard error, SLE: systemic lupus erythematosus, SLEDAI: Systemic Lupus Erythematosus Disease Activity Index | | | | | | |

| **Table S-16: Multivariate Logistic Regression Analysis for Studied Data As A Predictive Factor Of Response (50% Reduction At 6 Months) In CYC Group** | | | | | | |
| --- | --- | --- | --- | --- | --- | --- |
|  | **B** | **SE** | **p-value** | **Odds** | **95% CI** | |
| **Age** | -0.129 | 0.122 | 0.294 | 0.879 | 0.692 | 1.118 |
| **APL positivity** | 18.313 | 11603 | 0.999 | 89748602 | 0.000 |  |
| **Duration of SLE** | 0.087 | 0.131 | 0.509 | 1.091 | 0.843 | 1.411 |
| **Duration of LN** | 1.043 | 0.655 | 0.111 | 2.838 | 0.786 | 10.246 |
| **Class of LN** | 0.000 | 1.443 | 1.000 | 1.000 | 0.059 | 16.928 |
| **Activity index** | 0.176 | 0.297 | 0.553 | 1.193 | 0.666 | 2.135 |
| **Chronicity index** | -1.110 | 1.168 | 0.342 | 0.330 | 0.033 | 3.253 |
| **SLEDAI score** | -0.105 | 0.204 | 0.609 | 0.901 | 0.603 | 1.344 |
| APL: antiphospholipid antibodies, B: Regression coefficient, CI: Confidence interval, CYC: cyclophosphamide, LN: lupus nephritis, SE: Standard error, SLE: systemic lupus erythematosus, SLEDAI: Systemic Lupus Erythematosus Disease Activity Index. | | | | | | |

| **Table S-17: Multivariate Logistic Regression Analysis for Studied Data As A Predictive Factor Of Response (Complete Renal Response At 12 Months) In CYC Group** | | | | | | |
| --- | --- | --- | --- | --- | --- | --- |
|  | **B** | **SE** | **p-value** | **Odds** | **95% CI** | |
| **Age** | 0.054 | 0.067 | 0.421 | 1.056 | 0.925 | 1.205 |
| **APL positivity** | -0.357 | 0.788 | 0.651 | 0.700 | 0.149 | 3.279 |
| **Duration of SLE** | -0.018 | 0.054 | 0.742 | 0.982 | 0.883 | 1.093 |
| **Duration of LN** | 0.063 | 0.138 | 0.648 | 1.065 | 0.813 | 1.395 |
| **Class of LN** | 0.457 | 0.721 | 0.526 | 1.579 | 0.385 | 6.483 |
| **Activity index** | -0.005 | 0.153 | 0.975 | 0.995 | 0.738 | 1.342 |
| **Chronicity index** | -0.483 | 0.502 | 0.335 | 0.617 | 0.231 | 1.648 |
| **SLEDAI score** | 0.046 | 0.102 | 0.656 | 1.047 | 0.856 | 1.279 |
| APL: antiphospholipid antibodies, B: Regression coefficient, CI: Confidence interval, CYC: cyclophosphamide, LN: lupus nephritis, SE: Standard error, SLE: systemic lupus erythematosus, SLEDAI: Systemic Lupus Erythematosus Disease Activity Index. | | | | | | |

| **Table S-18: Impact Of Number of PLEX Sessions On Laboratory Response at Different Timelines** | | | |
| --- | --- | --- | --- |
| **Timeline** |  | **NUMBER OF SESSIONS** | |
|  |  | **Pearson Correlation** | **p value** |
| **3 months** | **Serum creatinine** | 0.042 | 0.77 |
|  | **Proteinuria in 24 hours urine** | -0.224 | 0.118 |
|  | **Serum C3** | 0.085 | 0.556 |
|  | **Serum C4** | -0.074 | 0.61 |
|  | **Platelet count** | -0.055 | 0.705 |
|  | **Serum LDH** | 0.216 | 0.131 |
| **6 months** | **Serum creatinine** | -0.091 | 0.532 |
|  | **Proteinuria in 24 hours urine** | -0.247 | 0.084 |
|  | **Serum C3** | 0.099 | 0.494 |
|  | **Serum C4** | 0.03 | 0.837 |
|  | **Platelet count** | 0.083 | 0.569 |
|  | **Serum LDH** | -0.1 | 0.488 |
| **12 months** | **Serum creatinine** | 0.005 | 0.975 |
|  | **Proteinuria in 24 hours urine** | 0 | 0.999 |
|  | **Serum C3** | -0.049 | 0.739 |
|  | **Serum C4** | 0.085 | 0.568 |
|  | **Platelet count** | -0.046 | 0.758 |
|  | **Serum LDH** | -0.051 | 0.73 |
| C3: Complement 3, C4: Complement 4, LDH: lactate dehydrogenase, PLEX: plasma exchange. | | | |

| **Table S-19: Impact of PLEX volume on laboratory response at different timelines** | | | | |
| --- | --- | --- | --- | --- |
| **Timeline** |  | **one volume (n=43)** | **1.5 volume (n=7)** |  |
|  |  | **Median (Range)** | **Median (Range)** | **p value** |
| **3 months** | **Serum creatinine** | 1(0.6-2.7) | 1.5(0.4-1.8) | 0.681 |
|  | **Urinary protein (gm/24hrs)** | 2 (1-3.3) | 2.3 (1.4-3.4) | **0.039** |
|  | **Serum C3** | 116(44-199.8) | 121(91-143) | 0.661 |
|  | **Serum C4** | 20(16-51) | 20(19-26) | 0.510 |
|  | **Platelet (x10^₃^)** | 152(98-177) | 154(132-169) | 0.827 |
|  | **Serum LDH** | 535(382-722) | 443(395-573) | 0.295 |
| **6 months** | **Serum creatinine** | 1(0.5-7.8) | 1.3(0.8-7.8) | 0.394 |
|  | **Urinary protein (gm/24hrs)** | 0.8(0.4-4.6) | 1(0.4-5) | 0.763 |
|  | **Serum C3** | 137(80-176) | 110(0.5-156) | **0.045** |
|  | **Serum C4** | 22(7.8-38) | 28.7(18-80) | 0.052 |
|  | **Platelet (x10^₃^)** | 214(80-240) | 209(40-232) | 0.364 |
|  | **Serum LDH** | 180(120-995) | 171(148-980) | 0.763 |
| **12 months** | **Serum creatinine** | 1(0.5-7.4) | 1(0.9-6.4) | 0.843 |
|  | **Urinary protein (gm/24hrs)** | 0.2(0.1-2.5) | 0.3(0.1-1.9) | 0.637 |
|  | **Serum C3** | 142(70-176) | 128(92-134) | **0.009** |
|  | **Serum C4** | 29(5-40) | 32(8-42) | 0.493 |
|  | **Platelet (x10^₃^)** | 250(40-287) | 242(90-265) | 0.553 |
|  | **Serum LDH** | 141(120-1200) | 143(126-870) | 0.891 |
| p value<0.05 means significant value, C3: complement 3, C4: complement 4, gm: gram, PLEX: plasma exchange. | | | | |
